# Recent perspectives on the association between osteonecrosis and bone mineral density decline in childhood acute lymphoblastic leukemia

**DOI:** 10.12703/r/10-57

**Published:** 2021-06-23

**Authors:** Jenneke E van Atteveld, Demi TC de Winter, Rob Pieters, Sebastian JCMM Neggers, Marry M van den Heuvel-Eibrink

**Affiliations:** 1 Princess Máxima Center for Pediatric Oncology, Heidelberglaan 25, 3584 CS Utrecht, the Netherlands

**Keywords:** Osteonecrosis, bone mineral density, childhood acute lymphoblastic leukemia

## Abstract

The attention to treatment-related toxicity has increased since the survival of children with acute lymphoblastic leukemia (ALL) has improved significantly over the past few decades. Intensive ALL treatment schedules including corticosteroids and asparaginase have been shown to give rise to skeletal abnormalities such as osteonecrosis and low bone mineral density (BMD), which may lead to debilitating sequelae in survivors. Although osteonecrosis and low BMD are different entities with suggested separate pathophysiological mechanisms, recent studies indicate that osteonecrosis is associated with accelerated BMD decline. Common underlying mechanisms for osteonecrosis and BMD decline are considered, such as an enhanced sensitivity to corticosteroids in children who suffer from both osteonecrosis and low BMD. In addition, restriction of weight-bearing activities, which is generally advised in patients with osteonecrosis, could aggravate BMD decline. This induces a clinical dilemma, since bone stimulation is important to maintain BMD but alternative interventions for osteonecrosis are limited. Furthermore, this recent finding of accelerated BMD decline in children with osteonecrosis emphasizes the need to develop effective preventive measures for osteonecrosis, which may include targeting BMD decline.

## Introduction

The increased survival of children with acute lymphoblastic leukemia (ALL) has led to a growing awareness of treatment-related toxicity^1^. Osteonecrosis and low bone mineral density (BMD) are two common skeletal toxicities of ALL and its treatment that may lead to important morbidity both during therapy and years thereafter^2^. In the past decade, our understanding of their pathophysiology and risk factors has increased, but effective preventive measures are still largely unavailable.

Although osteonecrosis and low BMD are different conditions, there is emerging evidence that these toxicities are associated and possibly even causally related^3,4^. However, limited knowledge is available about the mechanism of this relationship, which is pivotal when developing preventive strategies for symptomatic osteonecrosis and clinically important low BMD (i.e. BMD Z-score ≤–2, especially in the presence of low-trauma fractures). This review examines the relationship between osteonecrosis and BMD decline, common risk factors for both, and implications for treatment and preventive strategies.

## Osteonecrosis

In patients with osteonecrosis, the blood supply to the bones is insufficient to meet their demands, causing bone death^5^. This impaired blood supply may be caused by intravascular emboli (for example, resulting from a hypercoagulable state in patients treated for ALL^5^), increased bone marrow pressure, and/or direct blood vessel damage^6,7^. Clinically important symptomatic osteonecrosis occurs in about 1 to 8% of all patients treated for ALL^6^. Adolescents are most commonly affected (10 to 20%)^6^, and we recently showed in a large cohort of 1,470 children with ALL that the cumulative incidence of symptomatic osteonecrosis and the frequency of severe osteonecrosis (Ponte di Legno toxicity working group [PTWG] grade 4) are highest among patients aged 15 to 18 years (31.4%, 59% of those had severe osteonecrosis)^8^. In other hematological malignancies, this problem has been less extensively studied.

Symptoms are most commonly present in weight-bearing joints and range from slight limitations in the range of motion to severe pain and joint destruction^9,10^. Several classification systems for osteonecrosis exist, both clinical (based on symptoms) and radiological (based on magnetic resonance imaging [MRI] scans) as well as combined classifications. To facilitate comparisons of frequencies and severities across ALL treatment protocols, the PTWG has established a consensus definition and grading of osteonecrosis (Table 1), which is based on the severity of clinical symptoms and MRI abnormalities (involvement of weight-bearing bones, joint lines, or joint deformation)^11^. The Niinimäki classification allows radiological classification of osteonecrotic lesions at multiple sites^12^. The severity is based on the localization of the lesions (weight-bearing versus non-weight-bearing bones and epiphysis versus diaphysis/metaphysis), as well as the area of articular surface involvement (<30% versus ≥30%) and presence of joint deformation.

**Table 1.** Grading of osteonecrosis associated with treatment of childhood acute lymphoblastic leukemia according to the PTWG.

**PTWG grade 1**	Asymptomatic with findings only by MRI
**PTWG grade 2**	Symptomatic, not limiting, or only slightly limiting self-care activity of daily living. Lesions only outside joint lines in non-weight-bearing bones.
**PTWG grade 3**	Symptomatic, not limiting, or only slightly limiting self-care activity of daily living. Lesions in weight-bearing bones or affecting joint lines in non-weight-bearing bones.
**PTWG grade 4**	Symptomatic with deformation by imaging of one or more joints and/or substantially limiting self-care activity of daily living

MRI, magnetic resonance imaging; PTWG, Ponte di Legno toxicity working group

Symptomatic osteonecrosis most often occurs during the maintenance phase of ALL treatment^9^ but can already be present at ALL diagnosis^13^ and in rare cases even years after treatment cessation^14^. The main treatment-related risk factor is exposure to corticosteroids, especially when administered concurrently with asparaginase^8,15^. Patients who underwent hematopoietic stem cell transplantation (HSCT) are also at increased risk of developing symptomatic osteonecrosis^10^.

## Low bone mineral density and fractures

Children treated for ALL are at increased risk of BMD decline and consequent bone fractures^16,17^. A large national Dutch study showed that BMD of the lumbar spine was below normative values in children at ALL diagnosis and remained lower during treatment^17^. The 3-year cumulative incidence of symptomatic fractures was 17.8% in this cohort. Significantly lower BMD was observed in children with fractures versus those without. In a previous study including a subset of these patients, the fracture incidence was compared with that of healthy controls, which showed that the fracture rate in patients with ALL was 6 times higher^18^. In a well-characterized pan-Canadian cohort, the cumulative incidence of fractures was 36% (32.5% for vertebral fractures and 23.0% for non-vertebral fractures) from ALL diagnosis until 6 years of follow-up^16^. The peak annual incidence of vertebral fractures occurred at 12 months and of non-vertebral fractures at 24 months. Most importantly, every 1 standard deviation reduction in BMD Z-score at ALL diagnosis increased the risk of vertebral and non-vertebral fractures by 89% and 70%, respectively. At the same time, the skeleton has significant regenerative capacity, reflected by reshaping of vertebral bodies following vertebral fractures as well as an increase in BMD after ALL treatment discontinuation in most children^16,17,19^. It is not entirely clear whether low BMD during ALL treatment impacts bone health in the (very) long term, since longitudinal studies from the time of diagnosis with more than 10 years of follow-up are lacking. However, it is conceivable that a proportion of children with persistently low BMD may be at life-long increased risk of fractures and associated pain, vertebral deformity, and functional morbidity^20,21^. Hence, prediction of this subset of survivors is important. We recently published a prediction model for low BMD (Z-score ≤–1) and very low BMD (Z-score ≤–2) based on easily measured patient and treatment characteristics (including sex, attained age, height, weight, current smoking status, and previous cranial irradiation and abdominal irradiation), which correctly identified BMD status in most white adult survivors of childhood cancer^22^.

## Association between osteonecrosis and bone mineral density

In 2015, the association between symptomatic osteonecrosis and BMD decline during and shortly after ALL therapy was established for the first time^4^. We showed that at ALL diagnosis, lumbar spine and total body BMD were not significantly different in patients who subsequently developed symptomatic osteonecrosis versus those who did not. However, BMD decline over time was more pronounced in children with symptomatic osteonecrosis compared to children without symptomatic osteonecrosis, which started right after ALL diagnosis but became more substantial following osteonecrosis diagnosis. At cessation of treatment, this also led to a significantly lower BMD in patients with symptomatic osteonecrosis. Hence, we revealed that additional BMD decline in patients with symptomatic osteonecrosis started only after osteonecrosis occurrence. This study suggests that advised weight-bearing restrictions to avoid aggravation of osteonecrosis may accelerate BMD decline.

The results of a recently published study by Inaba *et al.*^3^ confirmed the association between osteonecrosis and BMD decline in 334 children with ALL who were prospectively screened for osteonecrosis by MRI. They found that knee osteonecrosis (59.3%) occurred more often than hip osteonecrosis (12.0%). Interestingly, they showed that the development of very low BMD (BMD Z-score ≤−2.0) was associated with osteonecrosis of the knee but not with osteonecrosis of the hip. The decrease in BMD Z-score was especially pronounced in patients with ≥30% epiphyseal osteonecrosis involvement. Inaba and colleagues did not separately analyze the course of BMD decline (before or after osteonecrosis occurrence) or the effect of osteonecrosis on BMD decline in patients with symptomatic versus asymptomatic osteonecrosis. This would be relevant because an observation that BMD decline is most evident after symptomatic osteonecrosis occurrence would support the idea that immobilization for osteonecrosis aggravates BMD decline. Since lumbar spine BMD alone was measured in the study by Inaba and colleagues, and not hip BMD (a site that is frequently affected by osteonecrosis), it is less conceivable that the reported general BMD decline was induced by destruction of the bone due to localized osteonecrotic lesions.

Hence, it remains unclear whether the most commonly used management for symptomatic osteonecrosis, i.e. weight-bearing restrictions, may aggravate the skeletal pathology in children with ALL. This is especially important since, although the application of these restrictions seems rational, the efficacy as a singular intervention to improve osteonecrosis symptoms has never been proven^23^. Alternatively, several common underlying mechanisms in children with osteonecrosis and low BMD might be considered, as discussed next.

## Common risk factors for osteonecrosis and low bone mineral density

Osteonecrosis and low BMD are considered different entities with a different pathogenesis; however, the co-occurrence of these side effects suggests that common factors may play a role in their pathophysiology (Table 2). Osteonecrosis (although uncommon) and low BMD can already be present at leukemia diagnosis, indicating that the leukemic disease itself (for example, due to enhanced bone resorption via cytokine release by lymphoblasts^24^) could play a role in the development of both complications^13,17^. Furthermore, the main treatment-related risk factor (i.e. corticosteroid exposure) is shared, so a common pathophysiology through enhanced sensitivity to corticosteroids may be considered^25,26^. Asparaginase increases dexamethasone plasma levels and may thus potentiate the detrimental effects of corticosteroids on the bone when administered concurrently^27,28^. In addition, especially PEG-asparaginase leads to hyperlipidemia, which is associated with osteonecrosis in children with ALL^29^. The association between hyperlipidemia and low BMD has not been established in this population, but, interestingly, studies in the general adult population do show such an adverse effect^30,31^. High-dose methotrexate may additionally modify the risk of osteonecrosis and BMD decline due to inhibition of bone formation and mineralization^6,32,33^, although it has not been identified as an independent risk factor for either toxicity in children with cancer. HSCT has been associated with an increased risk of symptomatic osteonecrosis, independently of corticosteroid dose^10^, and low BMD has been frequently described after HSCT, even in groups in which the majority had not been treated with corticosteroids^2,34^. In addition, direct radiation to the bone can lead to osteo(radio)necrosis due to damage of the microvasculature within and surrounding the bone, and cranial/craniospinal irradiation and total body irradiation have been associated with low BMD in survivors of childhood ALL^21,35,36^. Lastly, components of ALL treatment impair BMD by inducing mesenchymal stem cells to differentiate into adipocytes rather than osteoblasts, amongst other mechanisms^37^. Fatty infiltration into the bone marrow is also assumed to be associated with osteonecrosis development, although the main contributing factor is thought to be decreased blood flow to the bone due to microthrombi, which induces osteocyte death^6^.

**Table 2.** Common risk factors for osteonecrosis and low BMD in the context of pediatric ALL.

	ON	BMD↓
**Patient characteristics**		
Sex	**±** (female)	**?** (male)
Older age at ALL diagnosis	**+**	**±**
BMI	**±** (higher)	**+** (lower)
Caucasian race	**+**	**+**
**Disease factors**		
Leukemic disease	**±**	**+**
**Treatment factors**		
Corticosteroids	**+**	**+**
Asparaginase (+ GCs)	**+**	**+**
Methotrexate	**?**	**?**
Radiotherapy	**+**	**+**
HSCT	**+**	**+**
**Treatment-induced metabolic changes**		
Marrow adipose tissue	**+**	**+**
Hyperlipidemia	**+**	**?**

**Table:** 

**+**	Independent risk factor in childhood ALL
**±**	Conflicting evidence for this risk factor in childhood ALL
**?**	Associated with the outcome in other populations

ALL, acute lymphoblastic leukemia; BMD, bone mineral density; BMI, body mass index; GCs, glucocorticoids; HSCT, hematopoietic stem cell transplantation; ON, osteonecrosis; RT, radiotherapy

In addition, several common non-treatment-related risk factors for osteonecrosis and low BMD have been identified. Older age is the most important risk factor for osteonecrosis in children with ALL^6^ and has also been shown to increase the risk of BMD decline^17,38^. The role of sex in developing osteonecrosis has not been consistently shown; however, some studies in children with ALL have indicated that females are at increased risk for osteonecrosis^9,39^. A large study in 845 adult survivors of childhood ALL has shown that males are at increased risk of developing low BMD (Z-score ≤–1), although this association has not been shown during ALL therapy^21^. The effect of body mass index (BMI) on the risk of osteonecrosis is also opposite to its effect on low BMD: a high BMI has in some studies been associated with osteonecrosis (probably through the same mechanism as hyperlipidemia, since those conditions are highly correlated^40,41^), whereas low BMI or body weight has been associated with low BMD^17^. Finally, Caucasian children have been shown to be at increased risk for both osteonecrosis and low BMD^42-44^.

Variation in the occurrence of these serious side effects among similarly treated patients suggests a role for genetic susceptibility. Carriership of single nucleotide polymorphisms (SNPs) in, for example, the *MTHFR*, *MTRR*, and *VDR* genes has been associated with low BMD at ALL diagnosis and during ALL treatment^45,46^, and SNPs near glutamate receptor genes, in the *ACP1* gene, and in the *VDR* Fok I start site have been associated with the development of osteonecrosis^28,43,47^. Although genetic variations in the *VDR* gene led to an increased risk of both lower BMD and osteonecrosis, the exact SNPs were different.

## Interventions to overcome osteonecrosis and bone mineral density decline

Because of the possibility that BMD decline is accelerated by osteonecrosis occurrence, the development of adequate interventions for osteonecrosis becomes even more important, since this could prevent BMD decline to a certain extent. However, concomitant osteonecrosis and low BMD in pediatric ALL pose a challenging clinical dilemma. Weight-bearing restrictions to ameliorate osteonecrosis symptoms may be carefully considered to prevent the acceleration of bone density decline, as other treatment options for osteonecrosis and evidence for their effectiveness are limited. For this purpose, non-weight-bearing exercises such as swimming and cycling, under the supervision of a specialized physiotherapist, may be encouraged, as this perhaps enhances bone formation and stimulates muscle strength while limiting weight-bearing of the joints^4^. Bisphosphonates have been evaluated for pain reduction and prevention of articular collapse in patients with symptomatic osteonecrosis. Although small case-series have indeed suggested that bisphosphonates lead to pain relief and improved mobility, they failed to show a decline in the progression of joint destruction^48^. Whether early administration of bisphosphonates to children with asymptomatic osteonecrosis has the potential to mitigate disease progression remains unknown. Except for acute-phase reactions, no adverse events of bisphosphonate therapy were reported. However, the safety and efficacy of bisphosphonate administration during leukemia treatment needs to be demonstrated in preclinical studies and randomized controlled trials. Until then, their prescription should be restricted to compassionate grounds in selected patients. In case of low BMD, bisphosphonates are reserved for children with severe osteoporosis (BMD Z-score ≤–2 in the presence of clinically relevant fractures) and low potential for BMD restitution^26^.

In more advanced stage osteonecrosis (PTWG grade 3), several non-conservative treatment options to prevent articular collapse have been reported, such as core decompression (with or without mesenchymal stem cells), bone grafting, and osteotomies^49^. Unfortunately, evidence that these interventions prevent further joint damage remains limited, and a recent study in 85 children and young adults with osteonecrosis during ALL treatment suggested that core decompression does not delay or improve the rates of femoral head survival^50^. Furthermore, the timing of such interventions is difficult given the variable natural history of the progression of osteonecrosis. Preferably, these interventions should take place before any joint damage (before PTWG grade 4), although intervening too early in patients who would never experience joint collapse should be avoided, as about 60% of patients with symptomatic osteonecrosis show reversible symptoms^9^. The identification of patients at high risk for severe osteonecrosis could guide this decision, but, so far, only older age (about 15 to 21 years)^8,39,51^, multiple joints affected at diagnosis of osteonecrosis^51^, and the presence of bone marrow edema on MRI in some studies^52^ have been shown to increase the risk of progressive osteonecrosis.

Given the limited availability and efficacy of treatment options for osteonecrosis, some patients eventually require joint replacement^49^, which is an undesirable outcome, as these patients are young and prostheses have a lifespan of approximately 15 to 25 years^53^. Thus, strategies to identify patients at risk of severe, progressive osteonecrosis at an early stage, as well as strategies to prevent osteonecrosis, are preferable and should be pursued.

## Emerging preventive measures for osteonecrosis and bone mineral density decline

Several of these strategies have recently been explored or are currently under investigation. In 2012, Mattano and colleagues from the Children’s Oncology Group proved the first and only preventive measure for osteonecrosis thus far by showing that the administration of shorter pulses of dexamethasone reduced the risk of osteonecrosis in patients aged 10 to 21 years despite a higher cumulative dose and without compromising leukemia outcomes^39^. This simple scheduling modification was adopted in many ALL protocols^54^. However, our group showed that the protective effect of these shorter pulses on osteonecrosis development may be dependent on the therapeutic context and especially that the administration of concurrent intensified asparaginase (administered in current ALL protocols) could attenuate the benefit^8^. The ongoing British Osteonecrosis Study (BONES) will assess risk factors and specific radiological features that predict progression in those with (symptomatic) osteonecrosis (ClinicalTrials.gov Identifier: NCT02598401)^55^. Since in this study patients with ALL aged 10 to 24 are prospectively screened for osteonecrosis by MRI and for BMD decline by dual energy X-ray absorptiometry, it could potentially confirm whether BMD decline precedes and/or follows osteonecrosis. Furthermore, in assessing the association between osteonecrosis and BMD decline, stratification of patients with asymptomatic and symptomatic osteonecrosis could identify whether osteonecrosis itself (suggesting common risk factors), or interventions for symptomatic osteonecrosis (i.e. weight-bearing restrictions), are associated with BMD decline.

Janke *et al.* from St. Jude Children’s Research Hospital showed that hypertension might be a modifiable risk factor for osteonecrosis^7^ and initiated an ongoing randomized controlled trial investigating the effect of an antihypertensive drug (lisinopril) on osteonecrosis development (ClinicalTrials.gov identifier: NCT04401267). Furthermore, recent publications on the role of hyperlipidemia in the development of osteonecrosis^28,29,56^ and other toxicities^57^ prompted the design of a randomized controlled trial assessing the effect of omega-3 supplements on lipid levels and osteonecrosis occurrence in children and young adults with ALL treated according to the NOPHO (and now the ALLTogether) protocol, which is currently recruiting (ClinicalTrials.gov identifier: NCT04209244). The results of these trials are expected within the next few years and may hopefully identify an effective way to prevent osteonecrosis and thereby potentially low BMD as well.

Targeting BMD decline during ALL therapy directly is also an area of ongoing research. Adequate dietary calcium (200–1,100 mg/day) and vitamin D (at least 400 IU/day) is important^58^, and vitamin D and calcium supplementation has been shown to increase BMD in otherwise healthy children and adults with low vitamin D levels (25OHD levels <20 ng/ml)^59,60^. Whether vitamin D and calcium supplementation has a significant effect on estimates of bone strength in children with cancer at higher 25OHD thresholds remains unknown. Mogil *et al.* showed that low-magnitude, high-frequency mechanical stimulation significantly improved tibial trabecular bone content in a per-protocol analysis of childhood cancer survivors completing at least 70% of prescribed sessions^61^. The effect of this intervention on BMD parameters during ALL therapy is currently underway as part of the St. Jude Total Therapy XVII trial (ClinicalTrials.gov identifier: NCT03117751).

## Conclusion

The recently established association between osteonecrosis and accelerated BMD decline suggests that common factors may play a role in their pathophysiology. Furthermore, BMD declines complicate considerations about the treatment of osteonecrosis; weight-bearing restrictions should be carefully considered and supervised by an experienced physical therapist to minimize potential consequent BMD decline. The results of ongoing randomized controlled trials investigating the preventive effect of several agents on osteonecrosis and BMD decline are eagerly awaited.
